# Environmental exposure to pyrethroids and sperm sex chromosome disomy: a cross-sectional study

**DOI:** 10.1186/1476-069X-12-111

**Published:** 2013-12-17

**Authors:** Heather A Young, John D Meeker, Sheena E Martenies, Zaida I Figueroa, Dana Boyd Barr, Melissa J Perry

**Affiliations:** 1 Department of Epidemiology and Biostatistics, George Washington University School of Public Health and Health Services, Washington, DC 20037, USA; 2 Department of Environmental Health, University of Michigan School of Public Health, Ann Arbor, MI 48109, USA; 3 Department of Environmental and Occupational Health, George Washington University School of Public Health and Health Services, Washington, DC 20037, USA; 4 Department of Environmental Health, Rollins School of Public Health, Emory University, Atlanta, GA 30322, USA

**Keywords:** Aneuploidy, Uniparental Disomy, Endocrine Disruptors, In situ hybridization, Fluorescence, Pyrethroids

## Abstract

**Background:**

The role of environmental pesticide exposures, such as pyrethroids, and their relationship to sperm abnormalities are not well understood. This study investigated whether environmental exposure to pyrethroids was associated with altered frequency of sperm sex chromosome disomy in adult men.

**Methods:**

A sample of 75 subjects recruited through a Massachusetts infertility clinic provided urine and semen samples. Individual exposures were measured as urinary concentrations of three pyrethroid metabolites ((3-phenoxybenzoic acid (3PBA), *cis*- and *trans*- 3-(2,2-Dichlorovinyl)-1-methylcyclopropane-1,2-dicarboxylic acid (CDCCA and TDCCA)). Multiprobe fluorescence in situ hybridization for chromosomes X, Y, and 18 was used to determine XX, YY, XY, 1818, and total sex chromosome disomy in sperm nuclei. Poisson regression analysis was used to examine the association between aneuploidy rates and pyrethroid metabolites while adjusting for covariates.

**Results:**

Between 25-56% of the sample were above the limit of detection (LOD) for the pyrethroid metabolites. All sex chromosome disomies were increased by 7-30% when comparing men with CDCCA and TDCCA levels above the LOD to those below the LOD. For 3PBA, compared to those below the LOD, those above the LOD had YY18 disomy rates 1.28 times higher (95% CI: 1.15, 1.42) whereas a reduced rate was seen for XY18 and total disomy (IRR = 0.82; 95% CI: 0.77, 0.87; IRR = 0.93; 95% CI: 0.87-0.97), and no association was seen for XX18 and 1818.

**Conclusions:**

Our findings suggest that urinary concentrations of CDCCA and TDCCA above the LOD were associated with increased rates of aneuploidy. However the findings for 3BPA were not consistent. This is the first study to examine these relationships, and replication of our findings is needed before the association between pyrethroid metabolites and aneuploidy can be fully defined.

## Background

Sperm aneuploidy is a determinant factor of numerous reproductive problems, including infertility, congenital abnormalities, and pregnancy loss [1,2] and aneuploidy is estimated to affect approximately 5% of clinically recognized pregnancies and 0.3% of live births [1]. Aneuploidy results from the failure of chromosome pairs to separate properly during cell division. Toxicants may adversely affect germ cell DNA integrity [3]; however, the exact causes of aneuploidy are not well understood. A limited number of studies have identified risk factors for sperm aneuploidy, including chemotherapy [4], smoking [5], and increasing paternal age [6]. Sex chromosomes are particularly susceptible to aneuploidy and sperm that are sex chromosome aneuploid are viable and can result in offspring with fertility conditions such as Klinefelter Syndrome, Turner Syndrome [1,7-9].

Chemicals such as pesticides are known to disrupt hormone signaling that may interfere with recombination sequences [10], and prior epidemiologic studies have shown associations between occupational pesticide use and sperm aneuploidy (reviewed in 11). Pyrethroids (PYRs), a family of synthetic insecticides which are structurally based on the natural pesticide pyrethrum [11], have been identified as potential endocrine disruptors [10,12-14].

PYRs are the most frequently used home and garden insecticides in the United States, where an estimated 240 million applications of PYRs are made annually [15]. Consumption of food containing PYR residues, particularly vegetables and fruits, is the primary route of exposure in the US population [16]. Urinary metabolites of PYRs have been measured in a substantial proportion of the general population [17]. Despite wide scale use and human exposure, relatively little is known about the human reproductive health effects of environmental exposure to PYRs [16].

Although research findings have demonstrated the potential for adverse effects of PYRs on semen parameters and sperm DNA damage [18-23], the relationship between environmental exposure to PYRs and disomy in human sperm has not been investigated previously.

This study sought to investigate whether environmental exposure to PYRs was associated with altered frequency of sperm sex chromosome disomy among adult men.

## Methods

### Study subjects

Study subjects were a subset of 75 men from a parent study of 341 men assessing the impact of environmental exposures on semen quality. In the parent study, men aged 20–54 from couples seeking infertility evaluation at Massachusetts General Hospital (MGH) Fertility Center between January 2000 and May 2003 were eligible for inclusion. Approximately 65% of eligible men agreed to participate, and those declining participation primarily cited lack of time during their clinic visit as the reason for non-participation. Men who were presenting for post-vasectomy semen analysis and/or receiving treatment for infertility were excluded from the parent study. None of the men reported occupational exposure to pesticides or other agents known to be associated with semen quality. All participants also completed a self-administered questionnaire which collected demographic information, lifestyle factors, and medical and fertility history. Full details of the parent study have been described elsewhere [24]. Study participants provided urine and semen samples on the same day. As samples from the parent study had been used for other semen analysis research, eligibility for this study was based on availability of a urine and semen sample in the biorepository. Of the 341 men enrolled in the parent study, sufficient samples were available for 75 (22%). The parent study was approved by the Harvard School of Public Health and the MGH Human Subjects Committees. All subjects signed an informed consent prior to participation.

### Semen analysis

Subjects were asked to abstain from ejaculation for 48 hours prior to providing a semen sample at the clinic via masturbation. Samples were liquefied at 37ºC for 20 minutes before analysis. Analysis of the samples took place at the MGH Andrology Laboratory with andrologists blinded to exposure status. The volume, pH, color, and viscosity were determined for each sample. Measurement of the semen parameters has been previously described [24]. Sperm counts and percent motility were determined manually and then measured by computer-aided sperm analysis (CASA) using the Hamilton-Thorn Motility Analyzer (10HTM-IVO; Hamilton Thorne Inc., Beverly, MA, USA). A minimum of 200 sperm from 4 different fields were analyzed. Each sample was also prepared on two slides for morphological assessment using a Nikon microscope with an oil immersion 100x objective (Nikon Company, Tokyo, Japan). Sperm were scored using Tygerberg Strict criteria [25].

### Disomy analysis

Fluorescence in situ hybridization (FISH) analysis for the detection of sex chromosome disomy was performed by a single investigator blinded to exposure status. The procedures for this analysis have been described elsewhere [26]. Briefly, the FISH procedure was carried out for three chromosomes of interest: X, Y and 18 (autosomal control). While sex chromosome disomy (XX18, XY18, and YY18) was the primary outcome of interest, data were also collected on the frequency of 1818 disomy. A series of non-overlapping field images were taken for each prepared FISH slide using a fluorescence microscope and scored using custom MATLAB software. The software was designed to utilize scoring criteria for size and shape as reported by Baumgartner et al. [27]. A co-localization analysis allowed the software to identify sperm nuclei and the number of signals contained therein. The procedures and validation of this semi-automated scoring method have been reported previously by Perry et al. [28,29].

### Urine analysis

Urine samples were frozen at -20°C and mailed on dry ice to the Centers for Disease Control and Prevention Pesticide Laboratory. Analysts were blinded to study objectives and to the individual characteristics of the study samples. The PYR metabolites 3-phenoxybenzoic acid (3PBA), cis-3-(2,2-dichlorovinyl)-2,2-dimethylcyclopropane carboxylic acid (CDCCA) and trans-3-(2,2-dichlorovinyl)-2,2-dimethylcyclopropane carboxylic acid (TDCCA) were measured using a procedure previously described [30]. In summary, the samples were fortified with stable isotope analogs of the target analytes, underwent solid phase extraction, and were analyzed using high-performance liquid chromatography coupled with tandem mass spectrometry using turbo ion-spray atmospheric pressure ionization. The limits of detection (LODs) were 0.1 μg/L for 3BPA, 0.23 μ/L for CDCCA and 0.35 μg/L for TDCCA. 3PBA is a common metabolite of several PYRs including cyhalothrin, cypermethrin, deltamethrin, fenvalerate and permethrin; CDCCA and TDCCA are metabolites of cis-permethrin and trans-permethrin, respectively, in addition to other pyrethroids such as cyfluthrin and cypermethrin [31]. Specific gravity of the urine samples was measured using a handheld refractometer (National Instrument Company, Inc., Baltimore, MD, USA). Creatinine was measured photometrically using kinetic colorimetric assay technology with a Hitachi 911 automated chemistry analyzer (Roche Diagnostics, Indianapolis, IN, USA).

### Statistical analysis

Descriptive statistics for demographic and semen parameters were summarized using frequency distributions or means and standard deviations as appropriate. Semen parameters were dichotomized using the World Health Organization reference values for sperm concentration (<20 million sperm/mL) and motility (<50% motile sperm) and the Tygerberg Strict Criteria for morphology (<4% normal morphology) [25,32]. Due to the relatively low proportion and limited distribution of values above the LOD, pyrethroid metabolite concentrations were categorized as above and below the limit of detection (LOD). When the instrument did not detect an analyte in the sample, an imputed value equal to one-half the LOD was used. In some instances, samples with metabolite values below the LOD were detected by the machine. For these samples, the actual machine reading was used. Descriptive statistics for metabolites and disomy data were summarized as mean and standard deviation, geometric mean, median, and relevant percentiles.

Creatinine concentrations are commonly used to adjust for urine dilution, but some studies have shown that creatinine adjustment may not be appropriate for some organic compounds that can be conjugated by the liver [33]. Creatinine levels also vary by a number of other personal factors including sex [34,35]; age [36,37]; decreasing muscle mass [38,39]; diet [40]; and time of day or seasonality [41,42], so adjusting urine metabolite concentrations using specific gravity may be more appropriate [43]. Creatinine and specific gravity adjusted summaries as well as volume-based (unadjusted) values are presented.

Due to the large number of sperm being scored and the relatively low frequency of disomy, Poisson regression (SAS GENMOD procedure) was utilized to model the association between each of the disomy measures and the individual volume-based metabolite values in unadjusted and adjusted analyses. Histograms for all outcome variables were examined, and all displayed the shape of a classic Poisson distribution. The number of sperm scored and the number of disomic nuclei identified were summed for each subject with the unit of analysis as the individual subject. This approach offers the advantage that the use of the offset variable will standardize the number of sperm counted for each subject. The Poisson model was fitted using a disomy measure as a count of disomic cells (XX18, YY18, XY18, 1818, and total sex chromosome disomy) as the outcome variable, the natural logarithm of the number of sperm counted as the offset variable, the metabolite of interest as the independent variable along with age, smoking status, motility, log of sperm concentration, and specific gravity as covariates in the adjusted analyses The distribution of sperm concentration is typically non-normal and markedly positively skewed; therefore, log transformation is commonly used to deal with sperm concentration [44]. Age, motility, log of sperm concentration, and specific gravity were included as continuous variables, and smoking as never, former, and current. Abstinence time and morphology were also investigated as covariates but neither were significant predictors of disomy and did not alter the relationship between metabolites and disomy so they were excluded from the models. Metabolites were entered into the model as dichotomous variables categorized as above and below the LOD. Incidence rate ratios (IRRs) and 95% confidence intervals were calculated for each model. Because of concern that samples with creatinine concentrations >300 mg/dL or <30 mg/dL or specific gravity >1.03 or <1.01 may be too concentrated or too dilute to provide valid results [45], a sensitivity analysis was performed excluding these individuals. A sensitivity analysis was also performed excluding those individuals with fewer than 1000 nuclei scored. Statistical analysis was performed using SAS version 9.2 (SAS Institute Inc, Cary, NC).

## Results

Table 1 shows the demographic and semen parameter characteristics of the 75 study subjects. The men had an average age of 35.3 years and a mean BMI of 27.9 kg/m^2^. The majority of the men were white (90%) and non-Hispanic (96%). Most of the men (77%) had never smoked with only 4% (n = 3) current smokers. Of the 75 men, 11% (n = 8) had sperm concentrations <20 million/mL, 43% (n = 32) had <50% motile sperm, and 7% (n = 5) had <4% normally shaped sperm.

**Table 1:** Characteristics of the study population men (n = 75)

**Variable**	**Mean ± SD**
Age	35.3 ± 5.1
BMI (kg/m^2^)	27.9 ± 4.8
Semen concentration (in millions/mL)	110.1 ± 104.8
Semen motility (%)	54.2 ± 22.2
Semen morphology (% normal)	8.5 ± 3.8
**Race (n = 2 missing)**	**N (%)**
White	66 (90.4)
Black	2 (2.7)
Other	5 (6.9)
**Hispanic ethnicity**	
No	72 (96.0)
Yes	3 (4.0)
**Semen concentration**	
<20 million/mL	8 (11.0)
**Semen morphology**	
<4% normal	5 (7.0)
**Semen motility**	
<50% motile	32 (42.7)
**Abstinence time**	
< = 2 days	19 (25.3)
3-4 days	36 (48.0)
> = 5 days	20 (26.7)
**Smoking (n = 2 missing)**	
No	56 (76.7)
Current smoker	3 (4.1)
Former smoker	14 (19.2)

*SD* = Standard deviation.

*BMI*-Body Mass Index.

Table 2 provides a summary of the semen disomy results. A median of 6146 sperm nuclei were scored per subject. The observed median percentages of XX18, XY18, YY18, 1818, and total disomy were 0.3, 0.8, 0.3, 0.04, and 1.5 respectively.

**Table 2:** Number of nuclei scored and aneuploidy (n = 75)

**Variable**	**Mean ± SD**	**Median**	**25th percentile**	**75th percentile**
Nuclei (n)	7073 ± 4536	6146	3438	10,291
% X18	38.8 ± 8.4	41.4	34.2	45.1
% Y18	37.3 ± 9.2	40.4	33.4	43.5
% XX18	0.47 ± 0.49	0.31	0.18	0.57
% YY18	0.36 ± 0.30	0.28	0.19	0.43
% XY18	1.09 ± 0.76	0.83	0.58	1.43
% 1818	0.04 ± 0.03	0.04	0.02	0.06
Total Disomy %	1.92 ± 1.18	1.53	1.13	2.54

*SD* = Standard deviation.

Table 3 summarizes the unadjusted urinary pyrethroid metabolite as well as the specific gravity and creatinine adjusted concentrations. Of the 75 samples, 56% (n = 42) were above the LOD for 3PBA, 29% (n = 22) were above the LOD for CDCCA, and 25% (n = 19) were above the LOD for TDCCA. Although the percentage above LOD was higher for 3PBA, the median levels were slightly higher for TDCCA. The specific gravity and creatinine adjusted results were similar. All three pyrethroid metabolites were highly correlated (r = 0.92-0.97).

**Table 3:** Distribution of pyrethroid metabolite levels (μg/L) in urine (n = 75)

**Metabolite**	**Mean ± SD**	**Geometric mean**	**25%**	**Median**	**75%**	**90%**	**Range**
**Unadjusted**							
3PBA	0.57 ± 1.27	0.18	0.05	0.15	0.51	1.04	0.01-7.90
CDCCA	0.32 ± 0.72	0.12	0.06	0.12	0.25	0.56	0.001-4.30
TDCCA	0.64 ± 1.61	0.18	0.09	0.18	0.46	1.13	0.001-10.84
**Specific gravity adjusted**							
3PBA	0.65 ± 1.34	0.24	0.09	0.22	0.63	1.29	0.01-8.28
CDCCA	0.72 ± 1.73	0.15	0.07	0.15	0.33	0.57	0.001-5.25
TDCCA	0.38 ± 0.83	0.23	0.12	0.21	0.53	1.13	0.001-11.36
**Creatinine adjusted**	**(μg/g creatinine)**						
3PBA	0.50 ± 1.24	0.15	0.06	0.11	0.36	0.94	0.009-7.77
CDCCA	0.30 ± 0.90	0.10	0.04	0.09	0.20	0.37	0.001-6.84
TDCCA	0.56 ± 1.63	0.14	0.07	0.13	0.34	0.90	0.001-10.33

*SD* = Standard deviation.

*3PBA* = 3-phenoxybenzoic acid; LOD = 0.10 μg/L.

*CDCCA* = cis-dichlorovinyl-dimethylcyclopropane carboxylic acid; LOD = 0.23 μg/L.

*TDCCA* = trans-dichlorovinyl-dimethylcyclopropane carboxylic acid; LOD = 0.35 μg/L.

Table 4 provides the mean percentage of each of the evaluated disomies as well as total disomy stratified by detectable levels of pyrethroid metabolites. Table 5 provides the results of the Poisson regression models for each outcome adjusted for age, specific gravity and smoking when categorizing the metabolites was examined as above and below the LOD. CDCCA and TDCCA showed similar results when comparing rates of aneuploidy in those men above the LOD to those men below the LOD. The rates were increased by 7% to 30% across the aneuploidy outcomes with the highest risk seen for TDCCA and XX18 (IRR = 1.30; 95% CI: 1.17, 1.46) and the lowest risk seen for TDCCA and XY18 (IRR = 1.07; 95% CI: 1.00, 1.14) and no association with 1818. When comparing men with 3PBA concentrations above the LOD to those below the LOD, rates of YY18 disomy were 1.28 times higher (95% CI: 1.15, 1.42), whereas a lower rate (IRR = 0.82; 95% CI: 0.77, 0.87) was seen for XY18 and total disomy (IRR = 0.93; 95% CI: 0.87, 0.97). No association was observed between 3BPA and XX18 or 1818 disomy.

**Table 4:** Aneuploidy Mean ± SD by detection of pyrethroid metabolite levels (n = 75)

	**%XX18**	**%YY18**	**%XY18**	**%1818**	**Total disomy %**
**3PBA**					
≤LOD (n = 33)	0.44 ± 0.31	0.32 ± 0.81	1.14 ± 0.81	0.05 ± .0.03	1.90 ± 1.04
>LOD (n = 42)	0.49 ± 0.60	0.40 ± 0.37	1.04 ± 0.72	0.04 ± 0.03	1.93 ± 1.28
**CDCCA**					
≤LOD (n = 53)	0.46 ± 0.49	0.38 ± 0.33	1.05 ± 0.80	0.05 ± 0.03	1.88 ± 1.23
>LOD (n = 22)	0.50 ± 0.51	0.33 ± 0.23	1.18 ± 0.67	0.03 ± 0.03	2.00 ± 1.05
**TDCCA**					
≤LOD (n = 56)	0.44 ± 0.48	0.37 ± 0.33	1.05 ± 0.80	0.05 ± 0.03	1.85 ± 1.23
>LOD (n = 19)	0.56 ± 0.52	0.35 ± 0.23	1.20 ± 0.62	0.04 ± 0.03	2.11 ± 1.00

*SD* = Standard deviation.

*LOD* = Limit of detection.

*3PBA* = 3-phenoxybenzoic acid; LOD = 0.10 μg/L.

*CDCCA* = cis-dichlorovinyl-dimethylcyclopropane carboxylic acid; LOD = 0.23 μg/L.

*TDCCA* = trans-dichlorovinyl-dimethylcyclopropane carboxylic acid; LOD = 0.35 μg/L.

**Table 5:** **Adjusted**^**a **^**IRRs (95% CI) for XX, YY, XY, 1818, and total sex-chromosome disomy for pyrethroid metabolite levels above versus below the LOD**

	**XX18**	**YY18**	**XY18**	**1818**	**Total Disomy**
**3PBA**					
≤LOD (n = 33)	1.00	1.00	1.00	1.00	1.00
>LOD (n = 42)	1.00 (0.90, 1.10)	**1.28 (1.15, 1.42)**	**0.82 (0.77, 0.87)**	0.89 (0.65, 1.23)	**0.93 (0.87, 0.97)**
**CDCCA**					
≤LOD (n = 53)	1.00	1.00	1.00	1.00	1.00
>LOD (n = 22)	**1.22 (1.09, 1.36)**	**1.18 (1.05, 1.31)**	**1.07 (1.00, 1.14)**	0.89 (0.65, 1.21)	**1.12 (1.06, 1.17)**
**TDCCA**					
≤LOD (n = 56)	1.00	1.00	1.00	1.00	1.00
>LOD (n = 19)	**1.30 (1.17, 1.46)**	**1.19 (1.06, 1.34)**	1.01 (0.93, 1.08)	1.10 (0.84, 1.44)	**1.09 (1.04, 1.15)**

*IRRs* = Incidence rate ratios.

*CI* = Confidence interval.

*LOD* = Limit of detection.

*3PBA* = 3-phenoxybenzoic acid; LOD = 0.10 μg/L.

*CDCCA* = cis-dichlorovinyl-dimethylcyclopropane carboxylic acid; LOD = 0.23 μg/L.

*TDCCA* = trans-dichlorovinyl-dimethylcyclopropane carboxylic acid; LOD = 0.35 μg/L.

^a^Adjusted for specific gravity, age, smoking, motility, and log of sperm concentration.

Sensitivity analyses excluding individuals with creatinine concentrations >300 mg/dL or <30 mg/dL, specific gravity >1.03 or <1.01, or <1000 nuclei scored showed similar results (data not shown). To examine nonlinear relationships between the outcome and the predictor, generalized additive models with various types of spline smoothing were examined with no resulting improvement in model fit.

## Discussion

To our knowledge, this is the first study to evaluate associations between urinary PYR metabolites and human sperm disomy. Our results showed consistent increased risks ranging between 7-30% of XX, YY, XY, and total sex chromosome disomy among those with higher levels of PYR metabolites (CDCCA and TDCCA) after adjustment for potential confounders. The third metabolite (3PBA) also displayed a 28% increased risk for YY disomy, whereas there was a 16% decrease in the rate of XY disomy and a 7% decrease in total disomy.

One prior study investigated associations between ambient levels of the PYR fenvalerate among workers in a pesticide manufacturing plant and found significant increases in sex chromosome disomy in exposed workers (0.74%) compared to an internal (0.56%) and external (0.39%) control group [45]. The frequency of chromosome 18 disomy was also significantly higher in the exposed compared to the control groups (0.33%, 0.20%, and 0.12% respectively) [46]. Although the Xia et al. [46] study examined an occupationally exposed cohort of men and used air monitoring for exposure assessment, the magnitude of increased rates of chromosome disomies associated with PYR exposure are similar to the increases reported in our clinic-based sample of men whose main route of exposure was environmental. The distributions of PYR metabolite levels in the present study are comparable to those sampled from males in the National Health and Nutrition Examination Study (NHANES) 2001–2002 [17]. Unadjusted CDCCA and TDCCA concentrations were similar in the current study with median values below the limit of detection (0.1 μg/L and 0.4 μg/L), and 90th percentiles of 0.56 and 1.13 μg/L respectively, compared to 0.47 and 1.09 μg/L from NHANES 2001–2002. Unadjusted median and 90th percentiles for 3PBA were lower for the current study (0.15 and 1.04 μg/L respectively) compared to 0.30 and 1.55 μg/L from NHANES 2001–2002.

PYRs and their metabolites have been identified in multiple environmental media, including soil, aquatic organisms [47], sediment [48], and residues in food [49]. PYRs have also been detected in breast milk [50]. While there is a large body of literature on pyrethroid toxicology, not all biological effects and clinical signs and symptoms are completely understood, particularly in humans [51]. There is evidence from human reporter gene assays demonstrating the ability of PYRs to bind with and disrupt androgen receptors [52,53], and results from epidemiological studies suggest environmental exposure to pyrethroids is associated with changes in thyroid and sex hormone levels in men [54,55]. Because of their endocrine disrupting properties, the initial list of 67 chemicals being screened by the United States Environmental Protection Agency (USEPA) through its Endocrine Disruptor Screening Program includes six PYRs, selected based on their exposure potential. Binding to xenobiotic-sensing nuclear receptors, such as Pregnane X receptor (PXR), has also been documented [56]. PXR expression is found primarily in the liver, with some expression in testis and embryonic tissues in humans [57].

PYRs affect estrogen receptors in Sertoli cells [58] and some PYR forms appear to exert antiandrogenic effects by antagonizing the androgen receptor and affecting seminal vesicle weight [59]. Because pyrethroids are manufactured to kill target insects by affecting ion channel function, their actions on the activation and inactivation of voltage-gated sodium channels (VGSCs) and neuronal excitability are well understood, but less is known about pyrethroid effects on other target organs such as the testis [11,60]. Importantly, voltage gated sodium channels are present in human sperm and they have a role in supporting the regulation of motility [61]. Several animal studies show links between PYR exposures and sperm function, including sperm capacitation and semen quality [62] and sperm motility [63]. One study reported dose and time dependent effects of cyhalothrin exposure on structural and numerical chromosomal damage in primary spermatocytes in mice [64]. Whether VGSCs in sperm play a role in meiotic signaling that can affect nondisjunction requires investigation, as it could be the biological mechanism underlying the associations reported here.

Independent of the parent compound activities, evidence from in-vitro yeast assays suggests that CDCCA, TDCCA and 3PBA have anti-estrogenic activities and that 3-phenoxybenozic alcohol, a metabolic precursor to 3PBA, has anti-androgenic activity [14]. Additionally, 3PBA has been shown to have anti-androgenic activity in reporter gene assays [52,53]. Androgen suppression in rats has been demonstrated to affect the expression of a number of proteins involved in cell division in male germ cells [65]. Changes in the expression of these proteins and how they impact the incidence of sex chromosome disomy requires further study. There may be differences in the behaviors of individual pyrethroid metabolites that result in unknown enzymatic or proteomic impacts, including varying effects on the signaling for meiotic controls. In this study, the differences in the metabolic products that result in 3PBA may account for the observation that 3PBA was associated with an increase in YY disomy frequency and a decrease in XY disomy. Perhaps 3PBA or one of its precursors alters the activities of critical meiotic proteins that regulate the separation of chromosomes and chromatids. Further research into the potential influences of pyrethroids or their metabolites on meiotic control mechanisms is needed to better explain these findings.

There are limitations to the use of non-persistent pesticide exposure biomarkers. The metabolites measured in this study are not specific to a single pesticide. The correlations among the pyrethroid metabolites were high, making it difficult to distinguish possible mechanistic groupings. Biomonitoring of insecticide concentrations in urine is commonly used as an indicator of internal dose; however PYRs are rapidly metabolized and excreted [66] and a single urine analysis is not likely to be as robust as multiple measures in representing longer term exposure. Given that spermatogenesis occurs over approximately sixty days [67] and meiosis approximately occurs in the middle of the cycle, determining if exposure was present pre-meiosis is important to link exposures to errors causing nondisjunction. A prior study by Meeker et al. [68] showed that a single urine sample was reasonably predictive of levels over a three month average for metabolites of other non-persistent pesticides.

The men in this study were from a fertility clinic as opposed to the general population. For this to limit the external validity of these findings, the men who visited the fertility clinic would need to differ from men in the general population in a way that would alter response to PYRs, for which there does not appear to be evidence. While the men were members of subfertile couples seeking fertility treatment, over half had normal semen motility and approximately 90% had normal concentration and morphology.

Disomy frequencies in the current study are higher than in the prior occupational pyrethroid exposure study [46], as well as higher than previously reported ranges [69,70]. These studies did not include men seeking fertility consultation. A validated semi-automated method for disomy frequency determination was used in this study, whereas most prior studies have used manual scoring. One recent study among andrology clinic patients using automated methods found higher disomy rates than previously reported, with total sex chromosome disomy averaging between 1.3-1.5% total disomy; however, they found no differences between manual and automated estimates [71]. Our validation studies comparing automated to manual results for sex chromosome disomy in normozoospermic men from a fertility clinic found higher disomy estimates than previously reported in non-clinic studies, but results did not differ between manual and semi-automated methods [28,29]. Use of a validated semi-automated method for counting disomic sperm in this study was considered a methodological strength because it allowed for objective and reliable processing of a large number of samples.

Most causes of male factor infertility are unknown, but environmental exposures have been suspected as risk factors. Epidemiological studies have suggested an increased risk of aneuploidy associated with environmental exposures. The results reported here are preliminary in implicating PYRs as sperm aneugens, but point to the need to further study environmental exposures as risk factors for male factor infertility. Given that these exposures are widespread, questions remain as to what effects they might be having on population reproductive health.

## Conclusions

The results from this study suggest that men with detectable urinary concentrations of CDCCA and TDCCA have significant increases in almost all sex chromosome disomies compared to men with non-detectable urinary concentrations of CDCCA and TDCCA. Men with urinary concentrations of 3PBA above the limit of detection also have significantly increased rates of YY disomy, but have significantly reduced rates of XY and total disomy. This study was unable to determine how or why the effects of exposure would differ between the various sex chromosome disomy outcomes and across related PYR metabolites. This is the first epidemiologic study to examine the relationship between environmental exposure to pyrethroids and sperm sex chromosome disomy so findings require replication in future studies and in other populations.

## Abbreviations

BMI: Body mass index; CASA: Computer-aided sperm analysis; CDCCA: cis-3-(2,2-dichlorovinyl)-2,2-dimethylcyclopropane carboxylic acid; FISH: Fluorescence in situ hybridization; GM: Geometric mean; IRR: Incidence rate ratio; LOD: Limit of detection; MGH: Massachusetts General Hospital; NHANES: National Health and Nutrition Examination Survey; 3PBA: 3-phenoxybenzoic acid; PYRs: Pyrethroids; PXR: Pregnane X receptor; SD: Standard deviation; TDCCA: Trans-3-(2,2-dichlorovinyl)-2,2-dimethylcyclopropane carboxylic acid; USEPA: United States Environmental Protection Agency; VGSC: Voltage-gated sodium channels.

## Competing interests

The authors of this paper declare they have no actual or potential competing interests.

## Authors’ contributions

HY contributed to the statistical analysis and interpretation of data. JM contributed to the acquisition and interpretation of data specifically the semen analysis. SM contributed to the statistical analysis and interpretation of data as well as drafting and revising of the manuscript. ZF contributed to the interpretation of data. DB contributed to the acquisition of data specifically the pyrethroid metabolite analysis. MP contributed to the conception and design of the study, acquisition and interpretation of the data. All authors contributed to the drafting and revising of the manuscript and have read and approved the final manuscript.
